# A common network of residue-residue contacts underlies interactions between peptides and HLA class II complex

**DOI:** 10.1371/journal.pone.0341970

**Published:** 2026-04-10

**Authors:** Alexander E. Kister, Dmitry R. Leshchiner, Ilya Kister

**Affiliations:** Department of Neurology, New York University Grossman School of Medicine, New York, New York, United States of America; University of Pennsylvania Perelman School of Medicine, UNITED STATES OF AMERICA

## Abstract

The formation of a stable antigenic peptide-HLA II (p-HLA class II) complex is a critical early step in the adaptive immune response. In this work, we identify the residue-residue contacts that ‘anchor’ the peptide between the alpha and beta chains of HLA II and examine whether the anchoring residue-residue contacts are shared among different p-HLA II complexes. We hypothesize that there are similarities between the contact map of the alpha and beta chains of HLA II with CLIP (the fragment of the invariant gamma chain that binds to newly synthesized HLA II molecules) and the contact maps between the different alpha and beta chains of HLA II molecules with various antigenic peptides. To test the hypothesis, 81 diverse peptide-HLA II DR and DQ complexes, including CLIP-HLA II complex, were selected from the PDB database, and ‘Unified Residue Numbering’ was introduced for all complexes. The Unified Residue Numbering enables us to compare residue contacts across complexes for each position, e.g., to identify the position in peptides occupied by residues with the highest number of contacts with HLA II similar to CLIP position with the highest number of contacts in all structures, and to define characteristics of residues for each position. We also identified all ‘similar contacts’ in the analyzed structures. ‘Similar contacts’ are defined as contacts between same-numbered residues in the peptide and HLA II and CLIP with HLA II structure independent of physicochemical properties of residues involved in the contact. We found that in the 81 analyzed structures, 90% of contacts between peptide and the alpha chains were ‘similar contacts’, as were 80% of contacts between peptides and the beta chains. Thus, our approach to sequence alignment, which is based on alignment of similar contacts rather than similar residues, allows one to define the common network of residues that underlies the interactions between peptide and HLA II. We also consider several criteria for the specificity of antigen peptide loading into HLA II based on the structural and physicochemical characteristics of the residues involved in the ‘anchoring’ contacts. These data may be useful for refining existing computational algorithms that predict peptide interactions with HLA II complexes.

## Introduction

‘Loading’ of digested peptide fragments onto Human Leukocyte Antigen complex class II (HLA II) and the formation of stable peptide-HLA II complex (p-HLA II) for subsequent recognition by CD4 + T cells is a critical early step of the adaptive immune response [1–3]. HLA II is a heterodimer composed of two non-covalently associated alpha and beta chains, which form the peptide-binding groove (PBG). The genes that code for HLA II in humans are the most polymorphic genes in the human genome [4]. The high variability in the PBG region allows different HLA alleles to bind widely diverse repertoires of pathogenic peptides that can then elicit an effective immune response [5]. Conversely, erroneous binding of self-peptides to HLA II can lead to autoimmune diseases [6].

The importance of p-HLA II interaction for shaping the adaptive immune responses to both foreign and self-antigens can be illustrated by these two infectious and autoimmune diseases: in West Nile Virus (WNV) infection, the neuroinvasiveness of WNV, which determines infection severity, has been correlated with specific residues within the PBG of HLA-DQA1 and HLA-DRB1 molecules [7]. In narcolepsy type 1, an autoimmune reaction directed at hypocretin-secreting neurons of the hypothalamus only develops, with rare exceptions, in carriers of a specific HLA II allele – HLA-DQB1*06:02:01 [8].

Many computer algorithms have been developed to predict whether a given peptide will fit into a specific HLA [9–16]. The main principle of the AI-based peptide-HLA II binding prediction is to pool together binding data from various HLA alleles to train a single, unified model (“pan-specific approach”) [17,18]. The limitations of these approaches are that the quality and quantity of data used to train algorithms have a major impact on their forecasting efficiency. Another significant problem for machine learning-based methods arises from the difficulty of accurately modeling the ‘ambiguous’ binding process [19]. Because the ends of the peptide-binding groove (PBG) in HLA class II molecules are open, longer peptides can shift within the groove and adopt multiple binding registers. It is difficult for prediction algorithms to identify with high precision the boundaries of the binding region. HLA-II architecture allows many different peptide sequences to bind, but only at certain peptide positions do specific residue side chains engage in strong, position-specific contacts with the HLA-II molecule. Thus, the peptide binding process is relatively promiscuous, yet limited by certain structural requirements and other conditions such as the thermodynamic mechanism of complex formation. Deepening our understanding of the factors responsible for the binding of peptides and HLA II may help to improve the accuracy of the existing algorithms.

This study aims to elucidate the fundamental requirements for establishing interactions between the peptide and the HLA II complex. We utilize a structural approach based on the hypothesis that peptides that serve as antigens for HLA II retain many of the same contacts that underline the interactions between the CLIP (CLass II-associated Invariant chain Peptide), a fragment of the invariant gamma chain, and the HLA II complex. The rationale for this hypothesis is that CLIP serves as a kind of ‘generic lid’ on HLA II molecules, i.e., it is non-allele specific and binds to many different HLA II molecules. Therefore, CLIP-HLA II interactions may help identify the core contacts that anchor the peptide into the PBGs of different HLA II complexes. To enable the comparison of different structures, the ‘unified numeration of residues’ (URN) of the alpha and beta chains, as well as of the peptide, was created for two major types of HLA II – HLA II DR and HLA II DQ complexes. URN allows us to develop specific criteria for residue content within the most important positions in the peptide-HLA II complex that ‘anchor’ the peptide within the peptide-binding groove. These criteria include restrictions on residue volume, hydrophobicity, contact preferences of residue contact pairs, and peptide mobility for the residue positions that play a critical role in p-HLA complex formation; they can also be used to refine p-HLA II prediction algorithms.

### A brief overview of HLA class II structure and function

**Fig 1:**
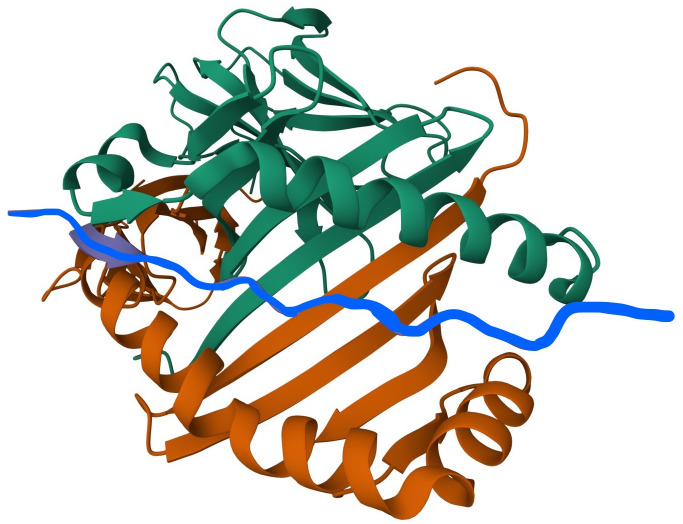
Peptide within the peptide-binding groove of the HLA II. Peptide/CLIP in the peptide binding groove of HLA II histocompatibility complex. The peptide binding groove is formed by the alpha chain (green) and the beta chain (orange). Peptide – CLIP (blue) is shown within the groove. The picture is based on PDB structure 3PDO.

HLA II molecules can be visualized as a ‘canyon’ inside which a peptide docks (Fig 1). One wall of the canyon is mostly formed by two helices of the α chain, and the other by four or five helices of the β chain. N-terminals of two chains form two beta sheets of four beta strands each and comprise the bottom of the canyon. A key paper on HLA class II 3-D structure is the crystal structure of HLA-DR1 complexed with an influenza peptide published in 1994 [20]. Numerous X-ray crystallographic analyses of HLA II molecules deposited in the Protein Data Bank (PDB) have confirmed that all HLA II structures have similar secondary and supersecondary structures, with some minor exceptions, such as the splitting of the one helix into two helices in beta chains. Tertiary structures may vary slightly among different HLA class II alleles, consistent with their primary function of presenting a large variety of processed antigens to CD4 + T cells.

### A brief overview of the invariant gamma chain sequence and functions

The gamma chain sequence consists of three parts: the N-terminal cytoplasmic tail, the transmembrane fragment, and the C-terminal extracellular segment, which includes a short region – about 15–20 residues – called ‘CLIP’. The CLIP fragment is located between the alpha and beta chains of the heterodimer, forming bonds with both chains and thereby maintaining a stable conformation of the PBG (Fig 1). CLIP closes the entrance to the groove like a lid, preventing the binding of intracellular peptides to the HLA II molecule [21–23]. In the absence of the gamma chain, an altered alpha-beta dimer conformation is observed, leading to improper peptide presentation and autoimmune reactions [24].

The CLIP fragment is divided into a central ‘core’ and the peripheral ‘Peptide Flanking Regions’ (PFRs). The CLIP core is usually nine amino acids long and has the most important contacts with the PBG [25]. The flanking regions vary in length and composition and can also interact with HLA II residues, influencing binding affinity, but the number of these contacts is much smaller than in the CLIP core.

## Materials and methods

A total of 81 diverse X-ray structures of p-HLA II complexes were selected from the PDB database. For each structure, the amino acid sequences of the alpha and beta chains and CLIP/peptide, as well as the atomic coordinates of all residues, were taken from the PDB structures. The structures were divided into two groups: 48 p-HLA II DR complexes and 33 p-HLA II DQ complexes. The p-HLA II DR complexes were compared with the ‘prototype‘ CLIP DR structure (PDB code: 3PDO), while the p-HLA II DQ complexes were compared with the prototype CLIP-HLA II DQ structure (PDB code: 5KSU).

The peptide sequences of the prototype structures 3PDO and 5KSU are present in S1 and S2 Tables, respectively. The corresponding alpha and beta chains sequences for both 3PDO and 5KSU are present in S3 Table.

### Residue-residue contacts between CLIP/antigenic peptide and HLA II alpha and beta chains

To calculate which residues form contacts and to assess the strength of contacts in the p-HLA II structure, we used Contact of Structural Units (CSU) software [26]. This approach, based on a detailed analysis of interatomic contacts (the nearest distance (Å) between the atoms of two residues; all putative hydrogen bonds; the contact surface area in Å²; and other characteristics), has been widely used for accurately calculating contacts in proteins [27,28]. The contact surface area is defined as the total area of an atom, residue, or molecular surface that is directly overlapping or is in close proximity to another atom or molecular surface within a complex, representing the physical interface where the chemical interactions occur. CSU provides insights into the extent of interaction between molecules and can be correlated with binding affinity and stability of molecular bonding.

### Protocol for developing a unified residue numbering for CLIP-HLA II and p-HLA II complexes

Step 1. As a starting point, a “prototype structure” is selected – the CLIP-HLA II structure from the Protein Data Base (PDB). The alpha and beta chain residues retain the same numbering as in the original PDB structure, while residues of the CLIP are renumbered sequentially, starting with the first residue assigned to position 1.

Step 2. Contacts between residues in the CLIP and in the alpha and beta chains of the HLA class II are then calculated. This contact map serves as the template for the ‘unified residue numbering’ of p-HLA II complexes.

Step 3. The preliminary step for the calculation of the contact map of p-HLA II complex is the sequence alignment of alpha and beta chains of p-HLA II complex with the alpha and beta chains in the prototype CLIP-HLA II complex. Overall, the alpha and beta chains of HLA class II form a homologous family with clear sequence and structural similarities that make their alignment straightforward across the different structures. As a result, residues in the alpha and beta chains of the p-HLA II complexes are assigned the same position numbers as residues in the alpha and beta chains of the corresponding prototype structures.

Step 4. The contact map with the unified numeration of alpha and beta chains of p-HLA II complex is then generated while the peptide’s residues provisionally retain their original numbering as in the PDB structure. Comparison of the contact maps of the p-HLA II and CLIP-HLA II structures allows us to determine the unified residue numbering for the peptide using the following algorithm:

a) A CLIP residue with a large number of contacts is selected on the contact map of the CLIP-HLA II structure, and the positions of the residues in the alpha and beta chains with which the selected residue in the CLIP forms contacts are determined.
b) The peptide residues with a large number of contacts are identified on the contact map in the p-HLA II structure. If one of these large-contact residues has all or most of its contacts with residues in alpha and beta chains at the same positions as the contacts of the residue in CLIP identified in above (a), then the peptide can be aligned with CLIP-HLA II structure using URN. If this condition cannot be satisfied, then the p-HLA II complex cannot be aligned with the prototypical CLIP-HLA II structure with URN.
c) If the p-HLA II contact map analysis reveals a ‘large-contact’ residue in the peptide that fulfills the above condition, then the residues’ positions in the peptide and CLIP are aligned, i.e., the peptide residue is assigned the same position number ‘X’ as the residue position that was identified in the CLIP-HLA II prototypical structure. These aligned pairs of contacts on the CLIP-HLA II and p-HLA II contact maps are called “similar contacts” (SC) if: 1. the peptide residue occupies the same position number as the corresponding residue in CLIP, and 2. residues in the peptide and CLIP interact with residues at the same positions in the HLA α and/or β chain, regardless of their chemical nature.

Step 5. The entire peptide sequence is then aligned as follows: the positions of residues in the peptide preceding the residue at position X are sequentially assigned positions X-1, X-2, etc., whereas the positions of residues in subsequent positions after the residue at position X are sequentially assigned position numbers X + 1, X + 2, etc.

Step 6. The contacts of the residue at each renumbered position in the peptide are compared with the contacts of the residue with the same-numbered position in the CLIP-HLA II complex to determine the presence of SC at each position. A necessary condition for accepting URN is the presence of at least one SC in at least 9 positions of p-HLA II. Furthermore, we require that at least 50% of all contacts in 9 peptide positions be similar to CLIP contacts in the aligned positions. The threshold of 9 positions was chosen because the length of the core peptide directly interacting with the binding groove is 9 amino acids long [29]. Failure to meet the condition means that the given p-HLA II complex cannot be represented in the same URN as the prototype CLIP-HLA II structure.

## Results

### Contact maps of CLIP and peptides bound to HLA II DR and DQ

#### Developing a unified residue numbering system for p-HLA II complexes.

Unified residue numbering for p-HLA II DR and DQ complexes was developed using the protocol described above based on two different prototype structures: PDB structure 3PDO for CLIP-HLA II DR and PDB structure 5KSU for CLIP-HLA II DQ structure. In both prototypes, CLIPs are the fragments of HLA class II histocompatibility antigen gamma chain (UniProt P04233-1: 103–120 structure 3PDO and 103–117 structure 5KSU).

Step 1. For CLIP, fragments of the histocompatibility HLA class II antigen gamma chain 103–120 and 103–117 (UniProt P04233-1) were selected, which correspond to the antigen peptides in the structures 3PDO and 5KSU, respectively. The positions of CLIP residues were renumbered as 1–18 in 3PDO and as 1–15 in 5KSU (see S1 and S2 Table). The number 1 residue – Pro (UniProt P04233-1 position 103) – is the first residue of UniProt P04233-1 sequence that is present in both 3PDO and 5KSU peptides.

Step 2. The contact maps for the prototype structures 3PDO and 5KSU were calculated as shown in S4 and S5 Table, column 3PDO, and in S6 and S7 Tables, column 5KSU.

Step 3. The residue positions in the alpha and beta chains of the 79 p-HLA II DR and DQ complexes were renumbered such that the corresponding residues have the same position numbers as in the prototype structures 3PDO and 5KSU (S3 Table).

Step 4. The contact maps using the URN of alpha and beta chains of all DR and DQ structures were calculated.

a) The DR CLIP residue at position 5 forms a large number of contacts: 9 contacts with the alpha chain, 4 – with the beta chain. The DQ CLIP residue at position 5 also forms a large number of contacts: 9 contacts with the alpha chain, 3 with the beta chain.
b) The analysis of contact maps for the 79 p-HLA II DR and DQ structures revealed one ‘high-contact’ position in each structure which had the largest number of contacts with residues in alpha and beta chains at the same positions as the contacts of residue in CLIP in position 5 in the prototype structures. The number of such contacts varied between 8 and 13 in DR complexes and between 7 and 10 contacts in DQ complexes.
c) Therefore, the ‘high-contact’ position in the DR and DQ structures identified in preceding paragraph (b) was assigned to position 5 – the high-contact position in the prototype structures, as per protocol Step 4, paragraph (b). The corresponding contacts are hereafter designated as “similar contacts” pursuant to paragraph (c) of the protocol.

Step 5. Per protocol, position 5 was used as a starting point for renumbering the remaining positions in the peptide sequences in the contact maps of p-HLA DR and DQ complexes. The positions of residues in the peptide preceding the residue at position 5 are sequentially assigned positions 4, 3, etc., whereas the positions of residues in subsequent positions after the residue at position 5 are assigned position numbers 6, 7, and so on.

Step 6. The contact maps of the p-HLA DR and DQ complexes were then compared with the respective prototype structures. At least one SC was found in at least 9 (and usually >9) positions, and in total, more than 50% of all contacts in these positions were SC. The total number of contacts and the number of SC for each position are shown in S8 Table (for DR structures) and S9 Table (for DQ structures). Overall, in the 81 analyzed structures, 90% of contacts between peptide and the alpha chains were SC (95% in DR, 84% in DQ structures), as were 80% of contacts between peptides and the beta chains in both DR and DQ structures. As the conditions for a URN were satisfied, the contact maps of all DR structures were combined into a unified contact map for DR structures (S4, S5 Tables), while the contact maps for DQ structures were combined into a unified contact map for DQ structures (S6, S7 Tables).

### Analysis of similar contacts in the DR complexes

CLIP residues at positions 3–14 in the CLIP-HLA DR structure form 45 contacts with the alpha chain residues and 38 contacts with the beta chain, for a total of 83 contacts (S4, S5 Table). To identify which of these contacts qualify as SC, we compared the contact maps of the prototype CLIP-HLA DR structure with contact maps of 47 p-HLA II DR complexes with UNR. In 40 out of 47 DR structures, the residues in each of the 12 peptide positions – positions 3–14 – had at least one SC as the respective residues in the CLIP-DR prototype structure. In total, the number of SC with the alpha and beta chains in the structures varied between 65 and 80 contacts (78–96% of all contacts in the prototype structure).

Note that in two structures (3L6F and 5JLZ) residues in one position, and in three structures (4MD0, 4MDU, and 6BIZ) residues in two positions were post-translationally modified. As a result, these residues were transformed into non-standard residues, which cannot be analyzed by CSU software. However, even without taking into account the contacts of the residues in these positions, the total number of SC varied between 59 and 74, i.e., the percentage of SC on these structures (between 71% and 89%) is approximately the same as in the structures in which all contacts were calculated for residues in each of the 12 positions of the core region. Therefore, even these 5 structures with ‘unaccounted’ contacts due to post-translation modification in 1 or 2 positions could be represented in URN.

The positions of residues in the alpha and beta chains that form SC with the peptide residues are listed in Table 1. In the HLA II DR complexes, 24 residues in the alpha chains form one or more SC: residues at positions 54, 62, 65, and 69 form 4 SC each; residues at positions 24, 53, and 72form 3 SC each; residues at positions 9, 52, and 76 form 2 SC each; and residues in the 14 remaining positions form 1 SC each. In the DR beta chains, 20 residues form one or more SC: the residue at position 71 forms 4 SC; residues in positions 13, 60, 61, 78, 81, and 85 form 3 SC each; residues in positions 28, 57, and 82 form 2 SC each, and residues in the 10 remaining positions form 1 SC each.

**Table 1:** Similar contacts (SC) of DR and DQ complexes.

Position	DR structures							DQ structures					
**in CLIP/**	**Contacts with alpha chain**			**Contacts with beta chain**				**Contacts with alpha chain**			**Contacts with beta chain**		
**Peptide**	**Position**	**secondary**	**% structures**	**Position**	**secondary**	**% structures**		**Position**	**secondary**	**% structures**	**Position**	**secondary**	**% structures**
	**numbers**	**structural units**	**with contacts**	**numbers**	**structural units**	**with contacts**		**numbers**	**structural units**	**with contacts**	**numbers**	**structural units**	**with contacts**
													
**3**	51	loop h1-h2	100	81	helix 4	61		**—**	**—**	**—**	**—**	**—**	**—**
	52	loop h1-h2	98	85	helix 4	100							
	53	loop h1-h2	100										
													
**4**	53	loop h1-h2	100	81	helix 4	100		52	loop h1-h2	53	81	helix 5	94
	54	loop h1-h2	87	85	helix 4	100		54	loop h1-h2	72	85	helix 5	88
													
**5**	7	strand 1	68	82	helix 4	100		9	strand 1	56	82	helix 5	100
	24	strand 2	100	85	helix 4	100		24	strand 2	100	85	helix 5	100
	31	strand 3	98	86	helix 4	98		31	strand 3	59	86	helix 5	31
	32	strand 3	100	89	helix 4	79		32	strand 3	94			
	43	strand 4	100					43	strand 4	84			
	52	loop h1-h2	100					48	helix 1	6			
	53	loop h1-h2	100					51	loop h1-h2	69			
	54	loop h1-h2	100					52	loop h1-h2	97			
	55	loop h1-h2	64					54	loop h1-h2	100			
													
**6**	24	strand 2	98	77	helix 3	98		9	strand 1	38	76	helix 4	0
	54	loop h1-h2	76	78	helix 3	100		24	strand 2	100	77	helix 4	100
				81	helix 4	100		54	loop h1-h2	47	78	helix 4	100
				82	helix 4	100					81	helix 5	94
											82	helix 5	100
													
**7**	9	strand 1	100	78	helix 3	98		9	strand 1	72	77	helix 4	22
	22	strand 2	100					22	strand 2	100	78	helix 4	100
	24	strand 2	89					24	strand 2	97			
	54	loop h1-h2	100					54	loop h1-h2	100			
	58	helix 2	98					58	helix 2	97			
	59	helix 2	41					62	helix 2	44			
	61	helix 2	37										
	62	helix 2	100										
													
**8**	9	strand 1	100	13	strand 1	100		9	strand 1	91	11	strand 1	100
	62	helix 2	98	26	strand 2	89		11	strand 1	84	13	strand 1	100
				71	helix 3	91		22	strand 2	81	26	strand 2	91
				74	helix 3	84		62	helix 2	100	28	strand 2	97
				78	helix 3	100					71	helix 3	56
											74	helix 4	100
											77	helix 4	19
											78	helix 4	100
													
**9**	62	helix 2	100	13	strand 1	80		61	helix 2	34	11	strand 1	100
	65	helix 2	26	71	helix 3	91		62	helix 2	100	70	helix 3	50
											71	helix 3	75
											77	helix 4	22
													
**10**	11	strand 1	71	11	strand 1	94		11	strand 1	28	9	strand 1	75
	62	helix 2	98	13	strand 1	68		62	helix 2	100	11	strand 1	100
	65	helix 2	100	28	strand 2	38		65	helix 2	100			
	66	helix 2	98	71	helix 3	47		66	helix 2	100			
	69	helix 2	81					69	helix 2	97			
													
**11**	65	helix 2	100	28	strand 2	66		65	helix 2	91	11	strand 1	19
	69	helix 2	98	47	strand 4	85		69	helix 2	100	47	strand 4	100
				61	helix 2	100					61	helix 2	100
				67	helix 3	100					67	helix 3	100
				70	helix 3	23					71	helix 3	69
				71	helix 3	94							
													
**12**	65	helix 2	100	60	helix 2	87		65	helix 2	100	60	helix 2	94
	68	helix 2	87	61	helix 2	100		68	helix 2	100	61	helix 2	100
	69	helix 2	100					69	helix 2	97			
	72	helix 2	57										
													
**13**	69	helix 2	100	9	strand 1	65		68	2	100	9	strand 1	53
	72	helix 2	100	57	helix 2	100		69	helix 2	100	37	strand 3	88
	73	helix 2	93	60	helix 2	89		72	helix 2	100	38	strand 3	25
	76	helix 2	91	61	helix 2	100		73	helix 2	100	53	helix 1	28
								76	helix 2	78	57	helix 2	100
											60	helix 2	78
											61	helix 2	100
													
**14**	72	helix 2	100	57	helix 2	84		68	helix 2	46	57	helix 2	75
	76	helix 2	89	60	helix 2	96		72	helix 2	79	60	helix 2	96
													

Legend to Table 1: Positions of residues in peptides (Column “Position in CLIP/peptide”) and in the alpha and beta chains (Columns “Position numbers”) are presented in URN. Column “Contacts with alpha (or beta) chains” shows the residue position numbers in the alpha or beta chains, involved in the contacts with a given peptide position, the next column shows secondary structural units in which these residues are found, and the Column “% structures with contacts” shows the percentage of the non-prototype structures in which the given SC is formed (relative to the total number of non-prototype structures).

### Analysis of similar contacts in DQ complexes

CLIP residues at positions 4–14 in the prototype CLIP-HLA II DQ structure form 43 contacts with the residues of the alpha chain and 42 contacts with the residues of the beta chain – a total of 85 contacts (shown in S6, S7 Table). Of the 32 p-HLA II DQ structures analyzed, complete data on residue coordinates in positions 4–14 were available for 28 structures, while in the remaining 4 structures, there was missing data on the coordinates of one peptide residue. Nevertheless, since SC were found in at least 11 positions even in structures with one missing position, URN was applicable for all analyzed p-HLA DQ structures. The total number of SC in the 32 DQ complexes varied between 72% and 89% of all contacts in the CLIP-HLA II DQ prototype structure.

In HLA II DQ complexes, 21 residues in the alpha chains formed 43 SC as the respective residues in the alpha chain of the prototype CLIP-DQ structure. Residues at positions 9, 54, 62, and 69 formed 4 SC each; residues at positions 24, 65, and 68 form 3 SC each; residues at positions 11, 22, 52, and 72 each formed 2 SC; and residues in 10 other positions formed a total of 10 SC. In the DQ beta chains, residues at 23 positions formed 42 SC: residues at positions 11 and 77 form four SC each; residues at positions 60, 61, 71 and 78 form three SC; residues at positions 9, 57, 81, 82 and 85 form two SC; and residues in 12 positions, formed a total of 12 SC (one per position).

### Comparison similar of contacts between DR and DQ complexe*s*

Residues at peptide positions 4–14 in the DR and DQ complexes have 78 and 85 SCs, respectively. A comparison of the SCs in the DR and DQ complexes revealed that 62 of the SCs are shared across DR and DQ structures (33 SC with alpha chains and 29 SC with beta chains). At least 2 common SC (with alpha and beta chains together) were found in each of the 11 positions, with 9 SC – the largest number of common SC was found in position 5 (S10 Table). More than 70% of SC are shared between DR and DQ, with at least 2 shared SC in each position. Thus, the sufficient criteria for representing the DR and DQ complexes within the same unified numeration system have been met.

### Peptide residues contacts with secondary structure units of alpha and beta chains

The basic units of the secondary structure of proteins are alpha helices, with approximately 3.6 amino acids per turn of the helix, and beta strands, which are straight, extended segments of a polypeptide chain. The irregular secondary structure unit – loop – is located between the helices and strands. The arrangement of secondary structure units in the alpha and beta chains of protein complexes is presented in S3 Table.

Table 1 shows which peptide residues form SC with which residues of the secondary structure units of the alpha and beta chains in the DR and DQ complexes. Comparison of the contacts of peptide residues with both the alpha and beta chain residues in the DQ and DQ complexes reveal certain similarities across structures. Most of the alpha chain peptide contacts at positions 4–8 in both DR and DQ structures are formed with residues in the loop between helices 1 and 2 and with strands 1, 2, 3, and 4. Starting from position 9 in the peptides, most contacts are formed with residues in helix 2. The secondary structures of the beta chains of the DR and DQ structures differ slightly. Helix 3 in DR complexes is divided into two helices in the DQ structures. Thus, residues at positions 4–6 have the greatest number of contacts with residues of helix 4 in DR complexes and with residues of helix 5 in the DQ structures. The contacts of residues at positions 8–14 of the peptides in both groups largely coincide and interact primarily with residues of strands 1–4 and helices 2 and 3 in DR structures, helices 2–4 in DQ structures.

### Analysis of residues of DR and *DQ* alpha and beta chains that form similar contacts

The residues of DR alpha chains form 45 SC with antigenic peptide residues at 24 positions, of which 15 are hydrophobic (S3 Table). Three of 24 positions (9, 52, and 76) form 2 contacts each, while the residues at positions 24, 53, and 72 (Phe, Ser, Ile) form 3 contacts each, and residues at positions 54, 62, 65, and 69 (Phe, Asn, Val, Asn) form 4 SC each (S4 Table). The ability of Asn to form multiple types of contacts, such as intermolecular hydrogen bonds and salt bridges, allows it to adapt to different structural contexts, potentially contributing to protein flexibility and dynamics.

Residues at 20 positions of DR beta chains form 38 SC, and 11 of these positions are occupied primarily by hydrophobic residues (S3 Table). Residues Glu, Asp, and Asn at positions 28, 57, and 82, respectively, form contacts with two peptide residues each, residues Phe, Tyr, Trp, Tyr, His, and Val at positions 13, 60, 61, 78, 81, and 85, respectively, form contacts with 3 residues each, while mostly Arg or Lys at position 71 form contacts with 4 residues (S5 Table).

The residues of DQ alpha chains form 43 SC with peptide residues at 21 positions, of which 11 are (primarily) hydrophobic (S3 Table). Four of 21 positions (11, 22, 52, and 72) form 2 contacts each, while the residues at positions 24, 65, and 68 (His, Val, His) form 3 contacts each, and residues at positions 9, 54, 62, and 69 (Tyr, Phe, Asn, Asn) form 4 SC each (S6 Table).

Residues at 23 positions of DQ beta chains form 42 SC, and 14 of these positions are occupied primarily by hydrophobic residues (S3 Table). Residues Tyr, Arg, His, Asn, and Leu at positions 9, 57, 81, 82 and 85, respectively, form contacts with two peptide residues each, residues Tyr, Trp, Lys, and Val at positions 60, 61, 71 and 78, respectively, form contacts with 3 residues each, while Phe and (mostly) Arg at positions 11 and 77, respectively, form contacts with 4 residues each (S7 Table).

### Physicochemical characteristics of peptides bound to HLA II

#### Hydrophobic and polar/charged residues in peptides.

Hydrophobicity scales are widely used as a general indicator for in-silico prediction of how well peptides bind to proteins, including MHC molecules [30]. Hydrophobicity scales assign a numeric value to each amino acid, reflecting its relative hydrophobicity. The sum of the residue hydrophobicity scores can be considered as the ‘peptide binding score’ for the given peptide. However, the results of these calculations are often ambiguous due to significant differences in the hydrophobicity scales. For example, according to the Kyte and Doolittle scale [31], the peptide in the 8CMB structure is very hydrophobic (the sum of hydrophobicity indices is 12.03), whereas according to the Jain et al. scale [32], this peptide is essentially non-hydrophobic (the sum of hydrophobicity indices is 0.29). Thus, the peptide binding score depends on the choice of the hydrophobicity scale.

To assess the ratio of hydrophobic and hydrophilic residues in peptides, we used a simplified quantitative residue comparison method, in which all hydrophobic residues are assigned a value of +1, and hydrophilic residues are assigned a value of −1. The sets of hydrophobic and hydrophilic amino acid residues are presented in S1 and S2 Tables. Gly residue with no acidic or basic group is considered ‘neutral’ (score of ‘0’). Applying this simplified scale to peptide fragments (positions 3–14) in DR complexes shows that the ratios of hydrophobic to hydrophilic residues are very wide-ranging. For example, in structure 4H1L, there are 8 hydrophilic and 4 hydrophobic residues, while in structure 6HBY, there are 9 hydrophobic residues, and in 8CMH structure, there are equal numbers of hydrophobic and hydrophilic residues. The number of Gly residues varies between 0 and 3 (S1 Table). Peptides in DQ complexes reveal a similar diversity, e.g., 10 hydrophobic residues in the structure 6U3N and 7 hydrophilic residues in the structure 2NNA (S2 Table).

Residues at the same position vary significantly in their physicochemical properties. For example, in 15 DR structures, position 12 is occupied by positively charged structures (Lys and Arg), while in 21 DR structures, the same position is occupied by hydrophobic residues (Ile, Leu, Val, and Ala). There are no conserved hydrophobic or hydrophilic positions. Only position 5 can be considered as an (almost) ‘conserved hydrophobic’ position, as hydrophobic residues occur in 47 out of 48 DR structures. In 8CMD structure, position 5 is occupied by Asn residue, and this single exception shows that the presence of a hydrophobic residue is not required in position 5. In certain contexts, Asn can be located within the hydrophobic part of a membrane protein and participate in interactions stabilized by hydrogen bonds between helices. This does not make Asn hydrophobic, but shows that its behavior is not always determined solely by its polarity.

Unlike DR complexes, hydrophobic residues at position 5 are found in only 22 structures out of 33 DQ complexes, while Glu, a polar, negatively charged amino acid, is found at this position in 9 structures (insulin and gliadin-related peptides, S2 Table).

### Volumes of CLIP and peptide residues

The volume of amino acid residues is an important factor influencing peptide binding with the alpha and beta chains. The first X-ray crystallographic studies of peptides within MHC class II binding groove revealed shallow depressions, called ‘pockets’, in the peptide binding groove in which the side chains of peptide residues are located [33]. The steric factor – residue volumes – influences the probability and efficiency of these binding interactions (i.e., how well certain residues can fit within the ‘pockets’).

The volumes of the amino acids can be classified as very small (60–90 Å³), small (108–117 Å³), medium (138–154 Å³), large (162–174 Å³), and very large (189–228 Å³) [34]. The volumes of individual residues and the total sum of residues’ volumes in a 12-residue peptide fragment (positions 3–14) in the HLA II DR structures are presented in S11 Table, and the volumes of individual residues and the total sum of residues’ volumes in an 11-residue peptide fragment (positions 4–14) in the HLA II DQ structures – in S12 Table. The CLIP peptide in the prototype structure has two very small residues (positions 3 and 8), two small residues (positions 9 and 10), one medium residue (position 14), and seven large residues (positions 4, 5, 6, 7, 11, and 12), and no very large residues. The total volume of 3–14 residues in the CLIP peptide is 1714.3 Å³. In various DR structures, the sum of volumes at 12 peptide positions – positions 3–14 – ranged from 1346.7 Å³ (5JLZ) to 1816.7 Å³ (4IS6). In DQ structures, the sum of volumes at 11 peptide positions – positions 4–14 – varied between 1170.6 Å³ (8VCY) and 1631.2 Å³ (3PL6). The distribution of residue volumes in any peptide position in URN is very diverse. A given position in URN can be occupied by residues with very small volumes in some structures and residues with large or very large volumes in other structures. For example, Ala (volume 88.6 Å³) and Trp (volume 227.8Å³) occupy position 12 in different DQ structures (4MAY and 1UVQ, respectively).

However, there exist specific characteristics in distribution of peptide volumes for each of thepeptide positions. For instance, position 10 is occupied by small or very small volume residues in 44 out of 46 peptides in DR structures, and by medium, small, or very small volume residues in 28 out of 30 peptides in DQ structures. For the 2 remaining peptides in each case, the volume is large. On the other hand, in position 5, the situation is opposite: in only 2 out of 46 DR peptides, position 5 is occupied by small or very small volume residues, but in more than half of the cases (25 out of 46), the peptide volume in position 5 is very large. These distributions are demonstrated in S11 and S12 Table. In each of the 23 distributions (for 12 positions in DR, 11 in DQ), ‘adjacent’ volume categories are found in half or more of all peptides (from 50% to 96%), e.g., for a peptide position 3 of DR structures, 34 out 49 structures contain a ‘very small’ or ‘small’ residue in that position. For DR, all possible pairs except “small/medium”, for DQ, all pairs except “large/very large” are present. That seems to indicate certain peptide residue volume preferences, specific to each position, and not identical between DR and DQ structures.

#### Conformational flexibility of a peptide.

Conformational flexibility of residues is the measure of how well amino acids can adapt to different spatial orientations and change shape, which is crucial for binding to other molecules. Conformational flexibility largely determines whether a given peptide can fit into the cavity between the alpha and beta chains of HLA II [35,36]. The analysis of conformational flexibility was carried out in accordance with the division of amino acid residues into three groups: ‘highly fluctuating’ (hf) residues (Pro, Ser, Ala, Gly, and Asp); ‘moderately fluctuating’ (mf) residues (Thr, Asn, Gln, Lys, Glu, Arg, Val, and Cys), and ‘weakly fluctuating’ (wf) residues (Ile, Leu, Met, Phe, Tyr, Trp, and His) [37]. In reality, the assessment of fluctuation requires a more nuanced evaluation as flexibility is related to many factors, such as hydrophobicity, position of the residue in the peptide, and physicochemical properties of the residue’s nearest neighbors [38]. For the analysis of peptides’ interactions with HLA II, where the peptide is “squeezed” into the canyon formed by the α- and β-chains, the choice of adequate indices of conformational flexibility is even more difficult. However, the above scale can be used as a first approximation to estimate the relative flexibility of residues in a peptide (S1 and S2 Tables).

In DR structures, in most positions of the core region (positions 3–14), the total number of hf and mf residues significantly exceeds the number of wf residues (S1 Table). Three exceptions exist: position 5, which is occupied by wf residues in 42 out of 48 structures (87.5%), while only one structure contains hf residue in position 5; then in position 11 and position 13, wf residues are found, respectively, in 50% and 54% of structures. More than 70% of hf and mf residues are observed in most positions in the core region (positions 4–14) in DQ structures, again with 3 exceptions: position 6, where in 70% of structures, wf residues are found, and positions 11 and 13, where wf residues are found in 42% of structures each (S2 Table). There appears to be a certain degree of similarity in residue flexibility position distributions between DR and DQ structures, while lower flexibility positions seem to be associated in some cases with positions with a larger number of SC.

### Contact Preference Scores for analysis of p-HLA II interface

‘Preference scores’ serve as a quantitative measure used to assess the favorability of contacts between peptide residues and HLA II DR residues. We used Contact Preference Scores (CPS) developed for large-scale computational docking [39]. The CPS values of all 210 unordered pairs of 20 residues can be conditionally divided into ‘strong’ (CPS from 80 to 100), ‘intermediate’ (CPS from 40 to 79), and ‘weak’ (CPS from 0 to 39). In the contact maps of the interaction of the peptides with the DR alpha and beta chains, preference scores are indicated for each pair of contacts (S4 and S5 Tables, respectively), as with DQ alpha and beta chains (S6 and S7 Tables, respectively).

In the prototype structure 3PDO, the HLA II DR alpha chains form 45 pairs of contacts, of which 8 (18%) pairs are classified as having strong CPS, 34 (75%) pairs as intermediate, and 3 (7%) pairs as weak. A preponderance of contact pairs in the intermediate CPS group, a relatively small number of ‘strong’ contact pairs, and a very small number of weak contacts, is observed for all structures. In 3PDO beta chains, out of 38 pairs of contacts, 6 (16%) pairs have strong CPS, 29 (76%) pairs are intermediate, and 3 (8%) pairs are weak. In DQ prototype structure 5KSU, the HLA II alpha chains form 43 pairs of contacts, of which 8 (19%) pairs are classified as having strong CPS, 32 (74%) pairs as intermediate, and 3 (7%) pairs as weak. In 5KSU beta chains, out of 42 pairs of contacts, 9 (21%) pairs are having strong CPS, 31 (74%) pairs are intermediate, and 2 (5%) pairs are weak.

To evaluate the preference of contacts for the structures, we calculated the average CPS of all contacts at each one of the peptide’s positions with alpha and beta chains in the p-HLA II DR complexes (S4 and S5 Tables, respectively) and DQ complexes (S6 and S7 Tables, respectively). Тhe highest average CPS of contacts with DR alpha chains is 71 in position 5, and the lowest one is 46 in position 10. For contacts with DR beta chains, the highest and lowest total average CPS is 65 (positions 5 and 12) and 45 (position 3). The next lowest average CPS is 48 (position 10). Тhe highest average CPS of contacts with DQ alpha chains is 64 in position 6, and the lowest one is 43 in position 10. For contacts with DQ beta chains, the highest and lowest total average CPS is 75 (position 12) and 44 (position 5). For position 10, the average CPS is 57. Presumably, higher CPS reflects the relative importance of residues at the given position for binding with alpha and beta chains.

We also calculated the average CPS of peptide-alpha chain contacts for each position in each structure. The average CPS for the prototype DR CLIP-HLA II structure was 60, while for the other peptides, the average CPS varied from 51 (structure 3L6F) to 64 (structure 8CMB). For the peptide-beta chain contacts, the average CPS score was 58 for the DR prototype structure and varied between 48 (structure 5NI9) and 65 (structure 8CMB) for DR p-HLAII structures. The average CPS for the prototype DQ CLIP-HLA II structure was 63, while for the other peptides, the average CPS varied from 46 (structure 7KEI) to 62 (structure 5KSV). For the peptide-beta chain contacts, the average CPS score was 60 for the DQ prototype structure and varied between 48 (structure 8VCY) and 64 (structure 9EJH) for DQ p-HLAII structures.

## Discussion

The novelty of our work is in the approach we use to compare diverse p-HLA II structures, which is not based on sequence alignment, graph theory, or machine learning techniques [40,41], but can be termed ‘contact-based alignment’. This approach makes it possible to develop a unified residue numbering system, whereby peptide residues in the same-numbered position share contacts with the residues in the same-numbered positions in alpha and beta chains across different structures (‘similar contacts’). As a result, we are able to delineate a network of contacts that are shared across p-MHC II structures not only within DR and DQ structures but also across DR and DQ alleles (S10 Table). Although anchoring residues that fit into MHC II ‘pockets’ are well described in the field of p-MHC II prediction [25], delineation of the common network of residues within a unified numbering system has not, to our knowledge, been presented previously.

The unified numbering system allows us to characterize each position within peptides with respect to their physicochemical properties, such as hydrophobicity, flexibility, polarity, and contract preference scores of residues that occupy the given position. These properties can be used as criteria for predicting whether a particular peptide will bind to a given HLA-II molecule. Our approach has some similarities with the MHCRule approach [16], but differs in its use of contact-based alignment and unified numbering system.

We propose that the question of whether an interaction is likely between peptide and the alpha and beta chains of HLA complex, can be resolved into two interdependent problems. First, can we predict whether a particular peptide has spatial and sequence characteristics that would allow it to fit into the peptide-binding groove formed by the alpha and beta chains? Second, if the steric characteristics are satisfactory, can we predict which arrangement of the peptide with the PBG will provide sufficiently stable contacts between the residues of the peptide and the alpha and beta chains?

To answer the first question of the compatibility of peptide and HLA II, we examined the properties of the peptide as a whole: the distribution of hydrophobic and hydrophilic residues, residue volumes, peptide sequence flexibility, and the contact preference (CPS) values. Restricting the ranges of ‘permitted parameters’ for these variables significantly delimits the range of peptides that can interact with HLA II. For example, the analysis of the hydropathy index of residues at positions 3–14 in the CLIP and peptide DR sequences revealed that both hydrophobic residues with positive hydropathy index and hydrophilic residues with negative hydropathy index can occupy these positions, but the absolute difference between the sums of positive and negative hydropathy counts does not exceed 6 in any structure (S1 Table). In DQ structures, however, it can go up to 9 (S2 Table). Another characteristic of peptides that bind with DR alpha and beta chains is that the residues at position 5 in these structures are always (with a single exception of 8CMD structure) hydrophobic (positive hydropathy index), including the partially hydrophobic residue Tyr. Importantly, the residue in position 5 of DR structures always has the largest number of SC with the alpha chain compared to the other positions of the peptide and a large average CPS value.

The constraints on the peptide total volume size follow from the calculated sums of residue volumes at the core region positions in the peptide sequence shown in S11, S12 Tables. The flexibility criterion of the peptide can also be used to restrict the selection of an antigenic peptide that will likely interact with HLA II. Based on the data presented in S1 and S2 Tables, it follows that the minimum number of highly fluctuating residues per peptide is 2 (except 3 cases - 4H26, 1H15, 3PL6), and the maximum number is 6 (except one case 5JLZ). When taken together with the moderately fluctuating residues, the total proportion of peptide’s’ residues that contribute to the plasticity of the flexible regions that are either highly or moderately fluctuating is no less than 50%.

If a putative peptide antigen meets most of the peptide compatibility criteria for binding to HLA II, the possibility of p-HLA II complex formation can be tentatively assumed, and the second question regarding the localization of the antigenic peptide within PBG is then considered. We propose that a peptide’s ability to bind to HLA II and its localization in PBG of HLA II depend on its ability to form a network of stable contacts with HLA II that is similar to the network of contacts observed in the 81 structures we analyzed here. Contact preference estimates can be used to determine the most likely residue positions in the Unified Residue Numbering System for the peptide/HLA II complex. Since the alpha and beta chain SC positions of each residue in the Unified Residue Numbering are known, several peptide localization options can be compared, and the optimal match can be selected as the most likely peptide localization in the peptide binding groove. Thus, it is possible to estimate whether a given peptide forms a complex with a given HLA II and to provide an outline of the tertiary p-HLA II structure based on the knowledge of peptide and HLA II sequence and secondary structure characteristics, without resorting to experimental methods for determining the three-dimensional structures of these complexes.

In summary, our work sought to identify the characteristics of peptides’ interactions with alpha and beta chains, which are shared across p-HLA complexes. We hypothesized that a network of contacts underlying p-HLA II interactions would be similar in its essential aspects to the interactions between CLIP and HLA II. The hypothesis was confirmed by the analysis of 81 diverse peptides-HLA II DR and DQ complexes. Thus, the general question of ‘Can the given peptide form a complex with HLA II?’ can be replaced with a much more specific one: ‘Can the given peptide form a network of residue-residue contact with the residues of HLA II, which is similar to the network of contacts shown in S4, S5, S6 and S7 Tables?

A limitation of our work is that our labor-intensive analyses were restricted to a diverse but relatively small number of peptide-HLA-II complexes, and therefore, it cannot be certain that these rules apply to all possible antigenic peptides that bind to HLA-II. However, it can be reasonably assumed that if the characteristics of the peptide match the conditions listed here, then it is highly likely that the peptide will form strong contacts with the HLA II complex. Indeed, the crystal structure of murine class II MHC I-Ab in complex with a human CLIP peptide has the same network of contacts as for human p-HLA II DR complexes (data not shown), suggesting that our results are generalizable.

The strength of our work is that the validity of the proposed criteria for peptide-HLA II interaction can be readily tested using AI-based generative computational biology methods on large peptide datasets. Validation of criteria for p-HLA II interactions may support their incorporation into existing computational algorithms for predicting peptide–HLA class II binding and improve the algorithms’ performance.

## Supporting information

S1 Table Unified residue numbering (UNR) for CLIP/Peptide residues in HLA II DR structures. All peptides were taken from different structures. Structures are considered different if the peptide sequences, or alpha or beta chains, differ in at least one position and if the structure contains another molecule, such as a T-cell receptor, which may affect peptide binding. The first column - “PDB code” – provides codes for p-HLA II DR and DQ structures, as per the PDB database. The structures marked with “*” are missing a residue in one or two positions of fragment 3–14 for DR structures or 4–14 for DQ structures, or the CSU software indicates a missing residue, as in the case of post-translational modification (such as citrullination of Arg residues in 4MD0, 4MD4, 6BIZ, 5JLZ structures and phosphorylation of Ser in 3L6F). Such nonstandard residues (modified monomers) are also marked with “*” in the Table. Also underlined are the residues that were present in the PDB peptide sequence, but their coordinates were unmodeled, so these residues were also missing with CSU software. The second column, “Peptide”, contains a description of peptides from PDB. The next column, “Num at 4 (PDB)”, shows a PDB number (residue number and chain ID) for the peptide residue in position 4 by URN (the first position present in all structures). The next column suggests a UniProt alignment for the peptide (if available). The column “Num at 4 (CSU)” shows the CSU number for the peptide residue in URN position 4. Columns “Unified numeration” contain the unified numeration of peptide positions 1–18. Residues in positions 3–14 in DR structures and 4–14 in DQ structures occupy all these positions in almost all structures and have contacts with residues of peptide-binding grooves formed by the α1 and β1 domains. These residues are considered to be ‘the core regions’ of peptides, and are enlarged and bolded. Columns ‘Hydrophobic’, ‘Charged/Polar’, and ‘Neutral’ list the count of hydrophobic, charged/polar, and neutral residues assigned to these residue groups. The list of residues included in each of the groups is provided as well. Residue Tyr, with a significant nonpolar region that attracts hydrophobic residues, is included in the hydrophobic group. The column “Hydrophobicity Count” presents a difference between the number of hydrophobic and charged/polar residues in a given structure. Columns “highly fluctuating”, “moderately fluctuating”, and “weakly fluctuating” list the number of residues in peptides assigned to these residue groups. The list of residues included in each of the groups is provided as well (e.g., the highly fluctuating residues are P, S, A, G, and D). The numbers of hydrophobic and fluctuating residues are calculated for core region residues only. At the bottom of the table, by each peptide position, the distribution of the number of structures with hf, mf, or wf residue in that position is demonstrated. At the bottom row, the % of wf cases is given for each position. In the side column, the % of hf, mf, or wf residues (for all positions together) is shown for each group.
(XLSX)

S2 Table Unified residue numbering (UNR) for CLIP/Peptide residues in HLA II DQ structures. Please see Legend to S1.
(XLSX)

S3 Table Sequences and secondary structures of alpha and beta chains in a Unified Residue Numbering for DR and DQ structures. The residue position in alpha and beta chains numbering corresponds to their numbering in the prototype structures 3PDO and 5KSU. The residue sequences for prototype structures are shown in the top line for each chain. The first and last position numbers of each helix and strand are shown in regular font. The position numbers of prototype residues in contact with peptide residues 3–14 are enlarged and bolded. Variations of residue content at these contacts in a given position are shown. For example, a contact position 54 in loop h1-h2 of DR alpha chain is occupied by C in the structure 3QXD but by F in all other structures. The hydrophobic residues are shown in italics. For example, the prototype DR alpha chain residue F in position 54 is hydrophobic, and shown in italics, while the variant residue C at the same position is not, and is shown in plain font. The position numbers of hydrophobic residues in the prototype structures are underlined. For example, position number 54 of the DR alpha chain is underlined, while position number 72 of the DQ alpha chain is shown in plain font, even though a variant residue I in that position is hydrophobic. Abbreviations: s1, s2… correspond to strand 1, strand 2, and so on; h1, h2… correspond to helix 1, helix 2, and so on. “Loop s1-s2” is a loop between strand 1 and strand 2. “Loop h1-h2 is a loop between helix 1 and helix 2, and similarly for the other loops.
(XLSX)

S4 Table The contact maps for CLIP/peptide interacting with HLA II DR alpha chains. The contacts map contains all similar contacts (SC) between CLIP/peptides and HLA II DR alpha chains. The first column contains position of the residue in the peptide in UNR (e.g., ‘p3’ in the third position in the peptide, p4 is the fourth position, etc.), while the second column contains the position of the residue in the alpha chain which makes contacts with the given peptide residue (e.g., residue in position 3 of the peptide makes contacts with residues in positions 51 and 53 of the alpha chain in HLA II DR complex). Column ‘Statistics of residues’ lists the counts of each residue at a given position in the alpha or beta chains. The data for each structure is presented in two columns (“cont Å²” and “Pref”) under the structure’s PDB code. The “cont Å²” column lists first the residue at the given position in the peptide (bolded). The post-translationally modified residues (citrullination of Arg, phosphorylation of Ser), missing with CSU software (so the contact data are not given), are marked with “*”. Also underlined are the residues that were present in the PDB peptide sequence, but their coordinates were unmodeled, so these residues were also missing with CSU software. In the lines below are listed the residues in the alpha or beta chain that form a ‘similar contact’ (SC) with the peptide residue. The CSA (Contact Surface Area) value given in Å² refers to the fraction of their accessible surfaces that become buried when they interact. The presence of hydrogen bonds (HB) is also indicated in this column. The “Pref” column presents the preference score (CPS) for the corresponding contact. At the bottom of the Table in these columns, are shown the values of the total average of CPS for a given structure (the bottom row: “Average CPS for structures”). The last column, “Average CPS (positions)” presents the value of the total average of CPS of all structures for each position.
(XLSX)

S5 Table The contact maps for CLIP/peptide interacting with HLA II DR beta chains. Please see Legend to S4.
(XLSX)

S6 Table The contact maps for CLIP/peptide interacting with HLA II DQ alpha chains. The Table is organized the same way as S4 Table. Column “Position Peptide”: p4–p14 in URN are shown for the core region. Other columns are organized identically to S4 Table.
(XLSX)

S7 Table The contact maps for CLIP/peptide interacting with HLA II DQ beta chains. Please see Legend to S6.
(XLSX)

S8 Table The number of all and SC contacts with alpha and beta chains for DR peptides positions. The DR structures are listed by rows. Peptide residues per position are listed in the same row for each structure. Peptide positions are given in columns; the alpha and beta chains’ contacts are listed separately. The post-translationally modified residues (citrullination of Arg, phosphorylation of Ser), missing with CSU software, are marked with “*”. Also underlined are the residues that were present in the PDB peptide sequence but whose coordinates were unmodeled, so these residues were also missing with CSU software. Number of contacts listed in 2 rows per structure: “Full” (number of all contacts) and “SC” (number of SC).
(XLSX)

S9 Table The number of all and SC contacts with alpha and beta chains for DQ peptides positions. The DQ structures are listed by rows.
(XLSX)

S10 Table Common URN of DR and DQ complexes. The Table sequentially compares the SC residues of the alpha and beta chains of DR and DQ. Positions of residues in peptides (Column: Position in CLIP/peptide) and in the alpha and beta chains are presented in URN. The positions of residues in the alpha and beta chains are enlarged and highlighted in bold if the same SC occurs in both the DR and DQ structures. The “Secondary structural units” column indicates the unit in which the residue is located at a given position. The “% Structure” column shows the percentage of non-prototype structures, in which a given SC is formed, to the total number of non-prototype structures.
(XLSX)

S11 Table Volumes of peptide residues in HLA II DR structures. For each residue, its volume in A³ and the group to which it belongs are indicated in the “Vol. A³” row and “Group” row (very small (vs), small (s), medium (m), large (l), and very large (vl), correspondingly). The total volume of all residues in the peptide is indicated in the column “Total volume”. The columns “very small”, “small”, “medium”, “large”, “very large” indicate the number of residues in each group. At the bottom of the table, by each peptide position, the distribution of the number of structures with a vs, s, m, l, or vl volume in that position is demonstrated. Below, the “most common pair” in this position (the pair of counts for two adjacent – like “vs” and “s” – volume categories that cover half or more of all samples) is listed. That same pair of numbers is also underlined in the summary Table (‘Counts for the residue types’) showing the number of structures with vs, s, m, l, or vl volume. At the two bottom rows, the total count of cases for that pair and the % of those cases (of all structures) are given for each position. At the side column, the total count of residues in each group (for all positions together) is shown.
(XLSX)

S12 Table Volumes of peptide residues in in HLA II DQ structures. Please see Legend to S12.
(XLSX)
